# Effects of Basic Amino Acids and Their Derivatives on SARS-CoV-2 and Influenza-A Virus Infection

**DOI:** 10.3390/v13071301

**Published:** 2021-07-04

**Authors:** Ivonne Melano, Li-Lan Kuo, Yan-Chung Lo, Po-Wei Sung, Ni Tien, Wen-Chi Su

**Affiliations:** 1 Graduate Institute of Biomedical Sciences, College of Medicine, China Medical University, Taichung 40402, Taiwan; ivonnemelano@yahoo.com; 2 Research Center for Emerging Viruses, China Medical University Hospital, Taichung 40402, Taiwan; ab801017@gmail.com; 3 Sinphar Pharmaceutical Co., Ltd., Sinphar Group, Yilan 269, Taiwan; beast_lo@yahoo.com; 4 School of Medicine, China Medical University, Taichung 40402, Taiwan; u105001405@cmu.edu.tw; 5 Department of Laboratory Medicine, China Medical University Hospital, Taichung 40402, Taiwan; t6719@mail.cmuh.org.tw; 6 Department of Medical Laboratory Science and Biotechnology, China Medical University, Taichung 40402, Taiwan; 7 International Master’s Program of Biomedical Sciences, China Medical University, Taichung 40402, Taiwan

**Keywords:** SARS-CoV-2, COVID-19, influenza A virus, lysine, arginine, disease prevention, antiviral therapy

## Abstract

Amino acids have been implicated with virus infection and replication. Here, we demonstrate the effects of two basic amino acids, arginine and lysine, and their ester derivatives on infection of two enveloped viruses, SARS-CoV-2, and influenza A virus. We found that lysine and its ester derivative can efficiently block infection of both viruses in vitro. Furthermore, the arginine ester derivative caused a significant boost in virus infection. Studies on their mechanism of action revealed that the compounds potentially disturb virus uncoating rather than virus attachment and endosomal acidification. Our findings suggest that lysine supplementation and the reduction of arginine-rich food intake can be considered as prophylactic and therapeutic regimens against these viruses while also providing a paradigm for the development of broad-spectrum antivirals.

## 1. Introduction

Coronavirus disease 2019 (COVID-19) is a global pandemic and has burdened the world since late 2019. With a reproductive number of around 3.28 [1], severe acute respiratory syndrome coronavirus 2 (SARS-CoV-2), the etiologic agent of COVID-19, has infected approximately 158 million people worldwide; while 3.29 million individuals have succumbed to the virus by April 2021 (Source: World Health Organization). Thus far, there are seven coronaviruses (CoVs) that can infect humans, and only three CoVs, including SARS-CoV, Middle East respiratory syndrome coronavirus (MERS-CoV), and SARS-CoV-2, cause severe symptoms and even death [2]. Recovered patients from SARS-CoV, which shares 82.45% homology with SARS-CoV-2 [3], were incapable of fully gaining back their lung function even after two years post-infection [4]. COVID-19, however, has a lower mortality rate yet shows similar clinical manifestations as SARS-CoV infection. Moreover, it has reports show that even after months of being cleared of SARS-CoV-2 viral antigen, people (called “long haulers”) were still experiencing the severe effects of COVID-19, such as extreme fatigue and brain fog [5]. Currently, no effective treatment against SARS-CoV-2 is available.

The influenza A virus (IAV), a causative agent of the common flu, is another respiratory virus that causes annual epidemics and recurring pandemics that threaten public health and the global economy. Though vaccines and antivirals against IAV are in development and production, its rapid evolution still poses a cause for concern [6]. Furthermore, it has a high rate of co-infection with SARS-CoV-2 [7]. Pre-infection of IAV augments SARS-CoV-2 infectivity, increases SARS-CoV-2 viral load, and induces more severe lung pathogenic changes [8,9].

Both of these enveloped viruses enter the cells via receptor-mediated endocytosis. Their virus surface proteins, SARS-CoV-2 Spike protein and IAV Hemagglutinin (HA), recognize and bind to the cell surface receptors ACE2 and sialic acid-containing receptors, respectively. The host cell then engulfs the virus by endocytosis. During endocytosis, the endosomes have low pH (pH 6.5-4.8) that triggers the viral attachment proteins to induce conformational changes of fusing with the endosomal membrane and proceed to uncoat and release its viral genome into the cytoplasm [10,11,12,13,14,15]. Another cell entry mechanism of SARS-CoV-2 is by plasma membrane fusion, wherein TMPRSS2, a cell surface protease, cleaves SARS-CoV-2 Spike to promote viral and cell membrane fusion; now permitting SARS-CoV-2 to release its viral genome into the cytoplasm [16,17,18].

**Figure 1:**
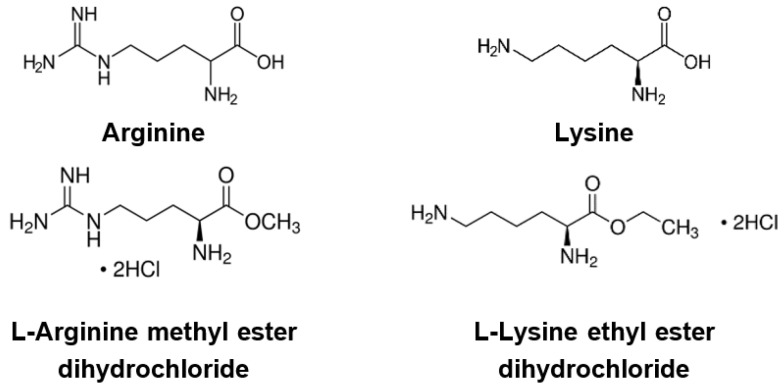
Chemical structure of the compounds.

A high mutation rate is a significant challenge in developing antivirals against RNA viruses. These mutations may benefit virus life cycles, such as evading immune response, increasing virus transmissibility, or enhancing pathogenesis. Hence, the need to discover broad-spectrum antiviral agents for current and future virus diseases is undisputed [19]. Previous research has shown that the amino acids arginine and lysine play roles in virus infection and replication [20,21,22,23,24,25,26]. Lysine, an essential amino acid, has long been prescribed against herpes simplex virus (HSV) infection [25,26,27]. A high lysine/arginine ratio has decreased HSV plaque formation in vitro [22,23,24]. Clinical trials have exhibited a reduction of recurrent HSV attacks, fewer healing days, and milder symptoms during a six-month period of L-lysine monochloride therapy [27,28]. Therefore, we investigated whether these basic amino acids could also affect SARS-CoV-2 and IAV infection. In this study, we used an in vitro system to evaluate the effects of lysine, arginine, and their derivatives, L-Arginine methyl ester dihydrochloride (Arg-ester), and L-Lysine ethyl ester dihydrochloride (Lys-ester) (Figure 1), on SARS-CoV-2 and IAV infection.

## 2. Materials and Methods

### 2.1. Cells

HEK293T cells were grown in DMEM (Gibco), supplemented with 10% FBS and antibiotics (100 U/mL penicillin G and 100 µg/mL streptomycin). Huh7 cells were grown in the same media but with added 1% non-essential amino acids. H1650 cells were grown in RPMI (Gibco), supplemented with 10% FBS (Hyclone) and antibiotics. A549 cells were maintained in an F-12K medium (Gibco), supplemented with 10% FBS and antibiotics. All cells were cultured at 37 °C and 5% CO_2_.

### 2.2. Plasmids and Viruses

pLAS2w.ACE2.Pbsd and pcDNA(TM)3.1(+)-2019-nCoV-S were provided by RNA Technology Platform and Gene Manipulation Core, Academia Sinica, Taiwan. pCAG.2-SARS-2-S-HA was constructed by amplifying SARS-2 Spike from pcDNA(TM)3.1(+)-2019-nCoV-S using Expand™ High Fidelity PCR System (Roche) with the following primers: 5′-actggctagcgccaccatgttcgtcttcctggtcctgctg-3′ (fwd) and 5′-actggaattcggtgtaatgcagcttcacgcccttc-3′ (rev), and inserted into NheI and EcoRI sites of HA-tagged vector pCAG.2. The construct was verified by Sanger sequencing.

The pseudotypes were produced by co-transfecting pcDNA3.1-spike (with truncation of C-terminal 18 amino acids), pCMA-d8.9, and pLAS3w-FLuc.puro using TransIT^®®^-LT1 Transfection Reagent (Mirus Bio) into HEK293T cells. Media were exchanged with complete media containing 1% BSA and 1X sodium butyrate at 18 h after transfection. The supernatant was collected every 24 h for 2 days, centrifuged at 3500 rpm for 5 min to remove cell debris and filtered with 0.45 μm filter. The aliquots were stored at −80°C. Primarily the A/WSN/33 strain (WSN33) of the influenza A virus (IAV) was used in this study.

### 2.3. Antibodies

For Western blotting, rabbit anti-ACE2, rabbit anti-IAV nucleoprotein (NP), and goat anti-rabbit IgG antibodies were purchased from GeneTex (GTX101395, GTX629633, and GTX213110-01). Rabbit anti-HA tag antibody was purchased from Millipore (04-902). Mouse anti-IAV matrix protein (M1) antibody was purchased from AbD Serotec (MCA401). For immunofluorescence staining, a mouse anti-IAV NP antibody was purchased from Abcam (ab20343). Alexa Fluor-conjugated secondary antibodies used for immunofluorescence were procured from Molecular Probes (Invitrogen). DAPI was purchased from Sigma-Aldrich.

### 2.4. Compounds

Most compounds were dissolved in water except bafilomycin A1 (in DMSO). The compounds used in this study are as following: L-Arginine, L-Lysine, L-Arginine methyl ester dihydrochloride, L-Lysine ethyl ester dihydrochloride, ammonium chloride (NH_4_Cl), and bafilomycin A1. All compounds were purchased from Sigma-Aldrich and used at the indicated concentrations in culture media.

### 2.5. Compound Treatment during Transductions

HEK293T cells were treated with the compounds in complete culture media for 1 h at 37 °C. After the 1-h incubation, SARS-2 S Vpp were spinoculated for 30 min onto the cells in the presence of the compounds and were then incubated at 37 °C overnight. Parallel cultures were incubated without Vpp for compound cytotoxicity assay. Media was exchanged for complete DMEM after the overnight incubation. At three days post-infection, cell viability and luciferase activity were measured as described below.

### 2.6. Cytotoxicity Assay

3-(4,5-dimethylthiazol-2-yl)-5-(3-carboxymethoxyphenyl)-2-(4-sulfophenyl)-2H-tetrazolium (MTS) (Promega) was diluted using DMEM without phenol red (Gibco) to a ratio of 1:9. Culture media was aspirated and the cells were incubated with MTS reagent at 37 ˚C until the MTS turned brown. Absorbance was then determined at 490 nm using Synergy™ H4 Hybrid Microplate Reader (BioTek). Values from control cells were calculated at 100% viability.

### 2.7. Luciferase Assay

Luciferase assay was conducted, using Bright-Glo™ Luciferase Assay System (Promega), according to the manufacturer’s instructions. Briefly, equal amounts of luciferase substrate were added to an equal amount of culture media per well. Cells were lysed for 2 min at room temperature then the luminescence was measured using the microplate reader. Values from control cells were calculated as 100% infectivity.

### 2.8. Immunoprecipitation Assay

HEK293T cells were transfected with either HA-tagged Spike or ACE2 plasmids using TransIT^®®^-LT1 Transfection Reagent and lysates were harvested 2-days post-transfection. 200 μg of Spike-expressing lysates and 200 μg of ACE2-expressing lysates were mixed and incubated for 2 h at 4 °C with the compounds. After incubation, the lysates were subjected to immunoprecipitation with Pierce™ Anti-HA Agarose (Roche Diagnostics) at 4 °C overnight. The immunoprecipitates were washed the next day with wash buffer I [50 mM Tris (pH 7.5), 150 mM NaCl, and 0.1% Triton X-100] and were analyzed by Western blotting.

### 2.9. Cell-Cell Fusion

Cell-cell fusion was completed through methods described previously [12]. H1650 effector cells were co-transfected with plasmids encoding Spike and GFP using Lipofectamine™ 2000 Transfection Reagent (Invitrogen), while H1650 target cells were transfected with ACE2. 2-days post-transfection, the effector cells were collected by adding 5 mM EDTA and overlaid onto the target cells for 4 h at 37 °C. Images were taken using a fluorescence microscope (Nikon Eclipse Ti-U).

### 2.10. Compound Treatment during IAV Infection

A549 cells were treated with the compounds in complete culture media for 1 h at 37 °C. After the 1-h incubation, the cells were infected with IAV, at MOI of 1, in the presence of the compounds using α-MEM (Gibco) with TPCK-Trypsin and were then incubated at 37 °C for 1 h. The cells were washed with PBS to remove excess IAV and then placed in the complete culture media with the compounds for 6 h at 37 °C. For binding assay, virus adsorption was done at 4 °C for 1 h. Cells were then washed with PBS to remove any unbound virus. For internalization assay, virus adsorption was also conducted at 4 °C for 1 h. After 1 h, the media were changed to complete culture media with the compounds and further incubated for 1 h at 37 °C. The cells were then washed once with PBS∙HCl pH 1.3 and twice with PBS only.

### 2.11. Western Blotting

To harvest cell lysates, M-PER™ Mammalian Protein Extraction Reagent (ThermoFisher) containing 50x protease inhibitor (Roche) was added to the cells. 4x Laemmli Sample Buffer (Bio-rad) and 2-mercaptoethanol (Aldrich) were added before boiling at 95 ˚C. Proteins were subjected to sodium dodecyl sulfate-polyacrylamide gel electrophoresis (SDS-PAGE) and transferred to a polyvinylidene difluoride (PVDF) membrane. For the blocking and dilution of antibodies, 5% skim milk in phosphate-buffered saline with Tween 20 (PBST) (Amresco) was used. Incubation of primary antibody was done overnight at 4 ˚C with constant shaking. After washing with PBST, goat anti-rabbit or anti-mouse was used as a secondary antibody. Immobilon Western Chemiluminescent HRP Substrate (Millipore) was used for visualization. Images were obtained using ImageQuant Las 4000 (GE Healthcare Life Sciences).

### 2.12. RT-qPCR

To extract the RNA, Trizol (Ambion) and chloroform were added to the samples. After centrifugation, the upper layer, which contains the nucleic acid, was collected. Glycogen was added and then proceeded with precipitation using isopropanol. The samples were washed twice with 75% ethanol, and pelleted RNA was resuspended in nuclease-free water. RNA yield was quantified using NanoDrop™ 2000c Spectrophotometer (ThermoFisher). cDNA synthesis was done using M-MLV Reverse Transcriptase (Invitrogen). The primers for reverse transcription are oligo (dT)20 and IAV-specific RT primer (uni-12; 5′-AGCAAAAGCAGG-3′). We followed the standard TaqMan method with the Universal Probe Library system (Roche) for quantitative PCR analysis. GAPDH was used as a normalization control for cellular mRNA and intracellular viral RNA. The primers and probes are the following: for IAV_NP segment: sense 5′-GATGGAGACTGATGGAGAACG-3′ and antisense 5′-TCATTTTTCCGACAGATGCTC-3′ with Universal Probe 59; and GAPDH: sense 5′-AGCCACATCGCTCAGACAC-3′ and antisense 5′-GCCCAATACGACCAAATCC-3′ with Universal Probe 60.

### 2.13. Acidic Vacuole Staining

A549 cells were seeded in a 24-well plate and treated with the compounds at 37 °C. After 1 h, 1 μg/mL acridine orange hydrochloride solution (Sigma) or 75 nM LysoTracker™ Red DND-99 (ThermoFisher) was added for 15 min at 37 °C. The cells were then washed twice with 1x PBS and observed by fluorescent microscopy. ImageJ software was used to calculate the fluorescence intensity of each cell.

### 2.14. Immunofluorescence Staining

A549 cells were infected with IAV at MOI 5 for 3 h at 37 ˚C. Cells were washed with 1x PBS. Fixation was done by adding 4% paraformaldehyde/PBS for 20 min. 0.5% Triton-X 100 (J.T. Baker) was used for permeabilization. 1% BSA in 1x PBS was used for blocking and dilution of antibodies. Cells were immunostained with anti-IAV NP antibody (1:100) and Alexa Fluor-conjugated antibodies (Invitrogen). DAPI was used to visualize the DNA. Images were acquired with a fluorescence microscope.

### 2.15. Statistics

The conventional Student’s *t*-test was used to determine statistical significances. The assays are representative of data from at least three independent experiments. Data are shown as means ± standard deviations (SD). Statistically significant values were considered when *p* < 0.05 (*), *p* < 0.01 (**) and *p* < 0.001 (***). A column scatterplot was generated by the PlotsofData web app, which also provided the 95% confidence intervals [29].

## 3. Results

### 3.1. Lysine and Its Derivative Attenuate SARS-CoV-2 Vpp Infection

**Figure 2:**
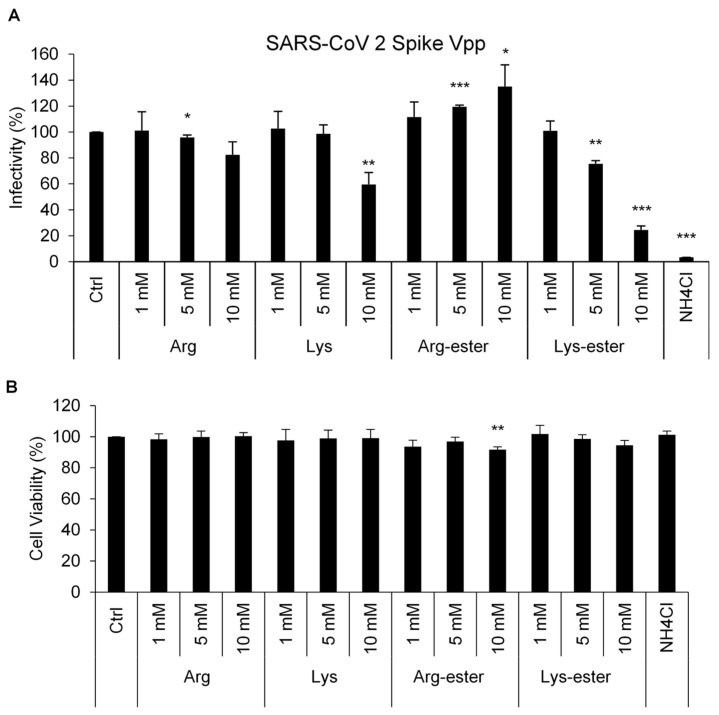
Compound effects on SARS-CoV-2 Spike Vpp infection in HEK293T cells. HEK293T cells were pre-treated with different concentrations of the compounds for 1 h and transduced with SARS-CoV-2 Spike Vpp for 1 h in the presence of the compounds in the media. (**A**) Infectivity and (**B**) cell viability were measured at 3 days post-infection using luciferase assay and MTS assay, respectively. The values represent the means ± standard deviation (SD) of data from three independent experiments. Ctrl: medium only. *, *p* < 0.05; **, *p* < 0.01; and ***, *p* < 0.001 compared with controls (n = 3).

Weak bases, like chloroquine analogs, have been investigated to impede viral entry by interfering with endosome acidification [30]. Given that arginine and lysine are basic amino acids, we hypothesized that these compounds may affect SARS-CoV-2 entering into cells. To prove this hypothesis, we utilized an established luciferase-based pseudotyped viral particle (Vpp) system for rapid quantitation of infected cells. These Vpps carry SARS-CoV-2 spike (S) protein and can mimic natural SARS-CoV-2 virus entry. Upon entering target cells, the luciferase reporter is expressed, and its intensity corresponds to the number of infected cells [12]. Human embryonic kidney 293 (HEK293T) cells were infected with SARS-CoV-2 Vpp in the presence of the compounds, and the cell infection rate was measured by the detection of luciferase activity (Figure 2A). As expected, treatment with NH_4_Cl, a lysosomotropic weak base that blocks virus entry [16,31,32,33], diminished SARS-CoV-2 Vpp infection. Among the four compounds tested, lysine and Lys-ester remarkably reduced Vpp entry in a dose-dependent manner. 10 mM of lysine and Lys-ester could inhibit 40% and 75% of viral entry, respectively. In contrast, 10 mM Arg-ester significantly increased Vpp infectivity (1.35-fold). Treatment and infection in Huh7 cells also resulted in a significant decrease in Vpp infection in lysine and Lys-ester-treated cells (Supplementary Figure S1).

Furthermore, to understand the cytotoxicity of these compounds, a cell viability assay was conducted. As shown in Figure 2B, only treatment with 10 mM Arg-ester showed significant cytotoxicity in HEK293T cells. These altogether suggest that lysine and Lys-ester control SARS-CoV-2-S-mediated infection at non-cytotoxic doses.

### 3.2. The Compounds Do Not Interfere the Interaction between Spike and ACE2

**Figure 3:**
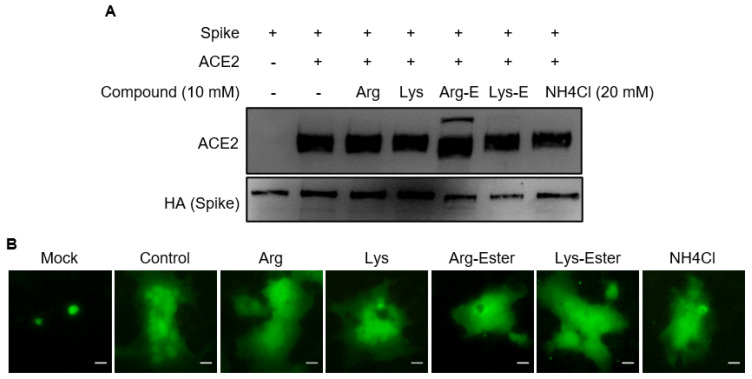
Effects of compounds on the interaction between SARS-CoV-2 Spike and ACE2. (**A**) HEK293T cells were transfected with either HA-tagged SARS-CoV-2 Spike or ACE2. Equal amounts of Spike-expressing and ACE2-expressing cell lysates were mixed, incubated with or without the compounds, and were used for immunoprecipitation assay using anti-HA agarose. The immunoprecipitates were resolved on SDS-PAGE and immunoblotted with anti-ACE2 or anti-HA antibodies. (**B**) Effects of compounds on SARS-CoV-2 Spike-mediated cell-cell fusion. Mock- or ACE2-transfected H1650 cells were co-cultured with spike and GFP-coexpressing H1650 cells for 4 h in the presence of the indicated compounds (10 mM) or NH_4_Cl (20 nM). Scale bar = 25 μM.

SARS-CoV-2 enters cells by binding its S protein to the ACE2 receptor protein of the host cell [16]. To evaluate whether the compounds affect the interaction between the S and ACE2, HA-tagged-S and ACE2 protein were separately expressed in HEK293T cells and their lysates were then used for immunoprecipitation assay in the presence of the compounds. The results indicated that no compound interfered with the interaction of Spike and ACE2 (Figure 3A).

After binding to ACE2, SARS-CoV-2 enters cells via fusion with cell membrane or endocytosis. We further investigated whether the compounds inhibited entry by interfering with the fusion of the viral membrane and the host membrane using a cell–cell fusion assay. In brief, we overlaid S protein and green fluorescent protein (GFP)-expressing human lung cancer H1650 cells on ACE2-transfected cells in the presence of the compounds. As shown in Figure 3B, large syncytia were formed despite compound treatment, implying that all tested compounds do not influence cell–cell fusion. These data collectively suggest that the compounds affect SARS-CoV-2-S Vpp infection by targeting a post-binding step.

### 3.3. Lysine and Lys-Ester Inhibit IAV Replication

**Figure 4:**
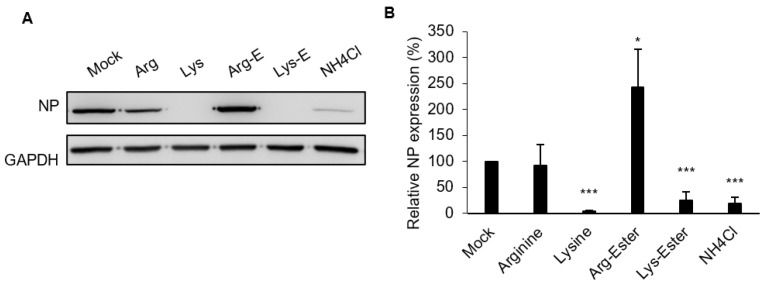
Compound effects on IAV infection in A549 cells. A549 cells were pre-treated with different concentrations of the compounds for 1 h and infected with IAV for 6 h in the presence of the compounds in the media. Cells treated with the medium were used as Mock control. (**A**) Cell lysates were harvested for Western blotting using anti-IAV NP antibody. GAPDH was used as a loading control. (**B**) Total RNA was extracted and used for RT-qPCR analysis. The levels of viral RNA were normalized with GAPDH RNA. The concentrations of the compounds were 10 nM, except for NH_4_Cl, 20 mM. The values represent the means ± standard deviation (SD) of data from three independent experiments. *, *p* < 0.05; and ***, *p* < 0.001 compared with controls (n = 3).

Next, we evaluated the effect of compounds on IAV infection due to it being the cause of outbreaks for decades worldwide and having a high prevalence of co-infection with SARS-CoV-2 [7]. As shown in Figure 4A, treatment of A549 cells with lysine and Lys-ester inhibited virus replication at 6 h post-infection (hpi), as evidenced by the reduction of nucleoprotein (NP) expression. This result was further verified in Figure 4B, wherein viral RNA was reduced to 4% and 25% by lysine and Lys-ester, respectively. Similar to the effect on SARS-CoV-2 Vpp infection, IAV viral RNA also increased in the presence of Arg-ester. These indicate that lysine and Lys-ester also have inhibitory effects on IAV infection, while Arg-ester has the opposite effect.

### 3.4. The Compounds Do Not Affect IAV Binding and Internalization

**Figure 5:**
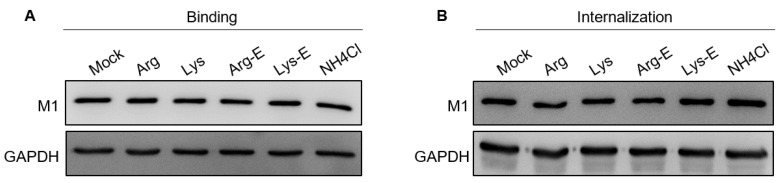
Compound effects on IAV binding and internalization. A549 cells were treated with the compounds for 1 h, then infected with IAV (MOI 5) at 4 °C for 1 h in the presence of the compounds. (**A**) After virus infection, the cells were washed with cold PBS to remove the unbound virus and then harvested. (**B**) Following virus infection, the cells were refreshed with a warm medium and incubated at 37 °C for an additional 1 h with the compounds. Cell lysates were harvested and analyzed by Western blotting with anti–M1 antibody. The concentrations of the compounds were 10 nM, except for NH_4_Cl, 20 mM. Mock: medium only.

The inhibition of SARS-CoV-2 Vpp infection by lysine and Lys-ester suggests that these compounds may affect virus entry (Figure 2A). For most enveloped viruses, virus entry steps include binding, internalization, endocytosis, and uncoating. To reveal the stage of the IAV replication cycle interfered with via the compounds, we first examined the effects of compounds on the binding and internalization of a virus. At 4 °C, the virus binds to the cell surface without internalization. At 37 °C, the virus can bind and be internalized into the cells [34]. Here, we detected virus particles by immunoblotting viral M1 protein, the most abundant structural protein of IAV particles. Compared to mock treatment, no significant change in the amount of bound (Figure 5A) or internalized (Figure 5B) M1 could be observed in the compound-treated cells, suggesting that the compounds most likely function at a step after internalization of virus particles.

### 3.5. The Compounds Do Not Reduce Endosomal Acidification

**Figure 6:**
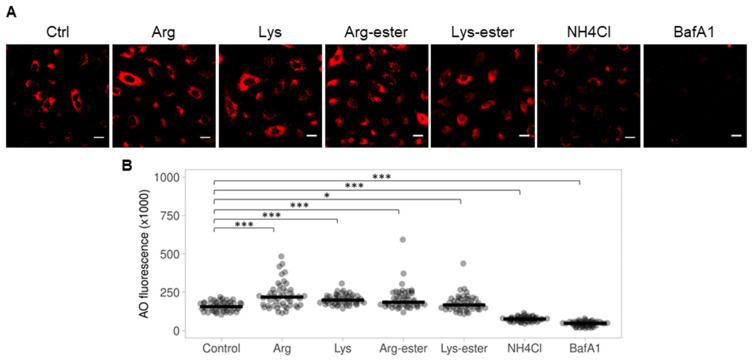
Acridine orange staining of A549 cells following compound treatment for 1 h. Cells were treated with the compounds (10 mM), NH_4_Cl (20 mM), or bafilomycin A1 (40 nM) for 1 h and stained with 1 μg/mL AO for 15 min. (**A**) Acidic vacuoles (orange-red) were observed using fluorescence microscopy. Scale bar = 100 μM. (**B**) Red fluorescence from 50 cells were calculated and plotted using ImageJ and PlotsOfData, respectively. Black horizontal lines represent the medians ±95% confidence intervals. *, *p* < 0.05; and ***, *p* < 0.001 compared with controls.

Soon after receptor recognition and attachment, the virus is endocytosed into the cell to proceed infection. SARS-CoV-2 and IAV require the acidic pH of the endosomes for a fusion of the viral membrane with the endosomal membrane to release its genome into the cytoplasm [11,13,14]. To examine whether the compounds reduce endosomal acidification, we performed acridine orange staining, which accumulates in acidic compartments where it fluoresces bright red [35]. Figure 6 illustrates that pre-treatment of cells with the compounds did not lessen the formation of acidic vacuoles. A marked increase in red fluorescence was observed after compound treatment (Figure 6B). However, cells treated with NH_4_Cl and bafilomycin A1, which are known to block endosomal acidification and are commonly used to demonstrate the requirement of acidic vacuoles in viral infectivity [36,37], exhibited lower fluorescence. In addition, we also performed LysoTracker Red staining to confirm our findings. LysoTracker Red staining demonstrated similar results with acridine orange staining (Supplementary Figure S2). These data altogether imply that the compounds did not inhibit virus infection by reducing endosomal acidification.

### 3.6. Lysine and Lys-Ester Reduce Nuclear Distribution of IAV

**Figure 7:**
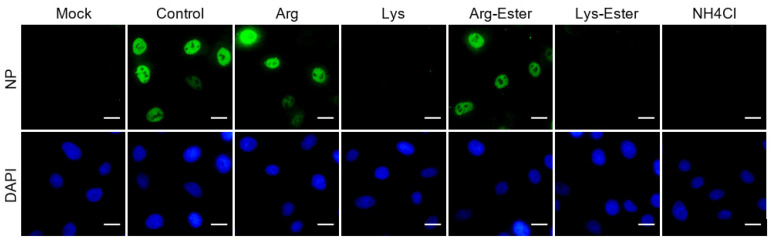
Compound effects on nuclear transport of IAV NP protein. A549 cells were treated with the compounds for 1 h and then infected with IAV (MOI 5) for 1 h at 37 °C in the presence of the compounds. After 3 hpi, cells were fixed and subjected to immunofluorescence staining with anti-NP antibody and Alexa Fluor 488 goat anti-mouse antibodies. Nuclei were counterstained with DAPI and cells were visualized with a fluorescence microscope. Scale bar = 20 μM.

Having demonstrated that the compounds might affect a step following endocytosis, we next examined if the inhibitory effects of lysine and Lys-ester were associated with virus release from the endosomes. Regarding the influenza virus, once the viral RNA is released into the cytoplasm, it is subsequently translocated to the nucleus. Thus, we evaluated the trafficking of the internalized virus by observing the nuclear localization of viral ribonucleoprotein (vRNP), which is composed of viral RNA, NP, and viral RNA-dependent RNA polymerase (RdRp). We found that lysine and Lys-ester treatment, similar to NH_4_Cl treatment, showed almost no NP signal in the nucleus (Figure 7). Combining all of the results, lysine and Lys-ester may most likely affect the stage after endocytosis, such as viral uncoating and nuclear import.

## 4. Discussion

There are three basic amino acids in the human body: arginine, lysine, and histidine. Considering that the side chain of arginine and lysine can resemble ammonia and their pKa values (lysine: pKa = 9.74, arginine: pKa = 10.76) are higher than histidine (pKa = 6), we focused on the effects of lysine and arginine on SARS-CoV-2 and IAV infection in the present study. We showed that lysine and Lys-ester can attenuate SARS-CoV-2 infection in the cell culture system (Figure 2A). Consistent with a previous study, IAV replication was also inhibited by lysine (Figure 4) [20]. Lysine therapy has long been recommended against HSV infection because it can suppress viral replication and inhibit virus yield [23,26,27,28,38]. HSV growth in both cell and tissue cultures that were supplemented with arginine was suppressed upon the addition of lysine [22]. A trial conducted on subjects with recurrent HSV infection also proved the efficacy of lysine treatment against HSV when the lysine group reported less recurrence, shortened healing time, and milder symptoms during six months of taking L-lysine monohydrochloride tablets while avoiding arginine-rich foods. [27]. These are just a few of the studies proving the efficacy of lysine as a prophylactic agent for HSV infection. Considering the prophylactic effects of lysine, a recent observational study was conducted using lysine therapy as prophylaxis for SARS-CoV-2 infection.

Among 30 medical professionals with daily face-to-face exposure to COVID-19 patients, all remain negative of the virus after taking 2000 mg lysine daily with required dietary restrictions (no caffeine, marijuana and arginine-rich foods) for 3 months, as compared to an average of two employees being infected every month prior to the study and to a public health department with similar capacity as their control group [39]. Our study validates that lysine has protective effects against SARS-CoV-2 and IAV infection. Thus, lysine supplementation may be considered as prophylaxis and therapeutic tool against these viruses.

Previous studies on HSV and IAV infection demonstrated that arginine can promote virus infection and replication [20,22,38]. In agreement, our data showed that pre-treatment of Arg-ester had a pronounced augmenting effect on both SARS-CoV-2 and IAV infection (Figure 2A and Figure 4). Cellular protease TMPRSS2 promotes SARS-CoV entry by cleaving S protein to activate it for membrane fusion and by cleaving ACE2 that leads to increased virus uptake [40]. As observed in Figure 3A, Arg-ester treatment caused the formation of a smaller ACE2, supposed to be the cleavage of ACE2. These findings suggest that Arg-ester may promote SARS-CoV-2 infection by inducing ACE2 cleavage.

The low pH of the endosomes plays a significant role in virus infection [11,13,37]. Initially, we thought that treatment with the basic amino acids would result in reducing proton concentration. However, our data revealed otherwise (Figure 6 and Supplementary Figure S2). The observed increase of acidic vacuoles in Arg-ester may be correlated with the rise in virus infectivity when treated with Arg-ester (Figure 2A and Figure 4). Nonetheless, further investigations are needed regarding the mechanism of lysine and Lys-ester against viral entry.

Most viruses enter cells primarily by receptor-mediated endocytosis. For SARS-CoV-2, the receptor-binding domain of its S protein recognizes and binds to the ACE2 receptor of the host cells and proceeds to endocytosis. Here, we used the SARS-CoV-2 Vpp system to highlight the effects of compounds on virus entry and discriminate the entry stage from other steps of the viral life cycle [17] (Figure 2A). Our findings demonstrate that the inhibitory effect of lysine and Lys-ester did not occur in viral attachment and penetration stages (Figure 3 and Figure 5), while it might function in uncoating or nuclear import (Figure 6 and Figure 7). Since SARS-CoV-2 Vpp infection does not involve nucleus translocation, we propose that the compound disturbed the step between endosomal fusion and uncoating. Several possibilities exist for the effect of lysine on the post-endocytic pathway. In the endosomes, viral proteins undergo processing to modulate viral and endosomal membrane fusion. Cleavage of both SARS-CoV-2 S and IAV HA proteins to S1/S2 and HA1/HA2 subunits, respectively, is necessary to activate their membrane fusion potential [41,42,43]. We speculate that lysine and Lys-ester may interrupt the interaction between fusion peptide and the endosomal membrane, thereby disabling the release of viral particles.

Another possible inhibitory mechanism of lysine is by altering the intracellular calcium levels. Deprivation of lysine in culture medium increased intracellular calcium levels and reduction of calcium efflux [44]. Moreover, viruses change calcium levels in support of their life cycle [45]. Introduction of SARS-CoV S protein induced a 3-fold upsurge of cytosolic calcium [46]. Likewise, a rise in cytosolic calcium was detected in IAV-infected cells [47]. Given that viruses can hijack calcium for their infection, we hypothesize that the addition of lysine could diminish the intracellular calcium levels and consequently hinder the virus infection.

Caveolae is another type of lipid raft that is composed of different proteins such as caveolin-1. More and more viruses, including two coronaviruses HCoV-OC43 and HCoV-229E, are proven to utilize caveolin-1-mediated trafficking following clathrin-mediated endocytosis [48]. SARS-CoV also exploits lipid raft during virus infection [49,50]. In this study, we did not investigate the involvement of lipid rafts in SARS-CoV-2 infections, but there is a report that NH_4_Cl inhibits virus infection by obstructing the intracellular trafficking of infectious viral particles from the endosomes to the caveosomes [31]. Perhaps lysine also hinders the trafficking of the virus to caveosomes.

Our primary focus is to study the effects of the amino acids in virus infection in vitro and delineate the potential mechanism of action. Further investigation of the detailed mechanism and evaluation of the inhibitory effect on other viruses are warranted for becoming broad-spectrum antivirals. Additionally, we did not determine the timing of treating the cells but instead treated the cells before and during virus infection. It will be crucial to determine whether adding the compounds after virus infection would yield the same effect.

## 5. Conclusions

Lysine and Lys-ester can prevent SARS-CoV-2 and IAV infection, particularly in the entry stage. In contrast to that, Arg-ester can potently boost infection of both viruses. It would therefore be beneficial to consider the nutrient intake of COVID-19 and flu patients. We recommend the inclusion of lysine supplementation in addition to a reduced arginine intake for the prevention and treatment of SARS-CoV-2 and IAV infections.

## Data Availability

The data presented in this study are available in this article and on request from the author and corresponding author.

## Supplementary Materials

The following are available online at https://www.mdpi.com/article/10.3390/v13071301/s1, Figure S1: Effect of compounds on SARS-CoV-2 Spike Vpp infection in Huh7 cells, Figure S2: LysoTracker Red staining of A549 cells following compound treatment for 1 h.
