# Supplementary material for: Effects of Basic Amino Acids and Their Derivatives on SARS-CoV-2 and Influenza-A Virus Infection
Source: Viruses. 2021 Jul 4;13(7):1301. doi: 10.3390/v13071301 (PMC8310019; doi:10.3390/v13071301)
Supplement: Supplementary file 1 [file viruses-13-01301-s001.zip › viruses-1240724-supplementary.pdf]

**Supplementary Figures**

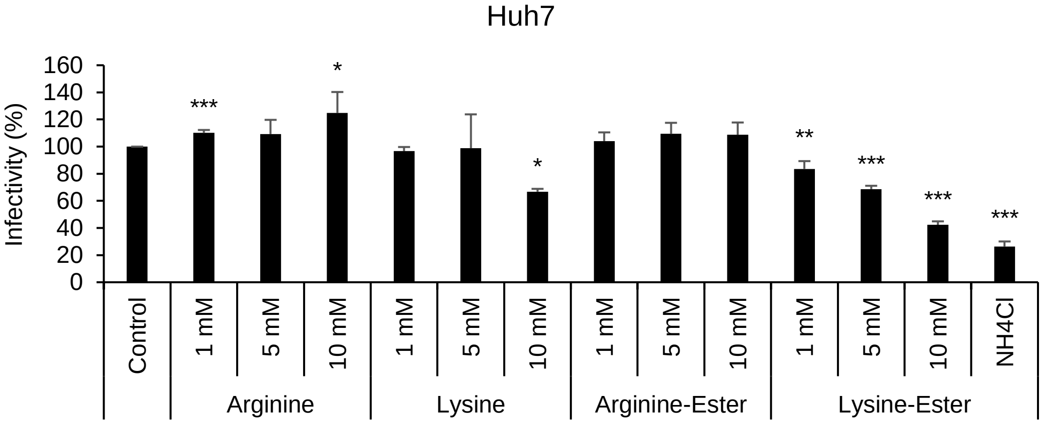


Supplementary Figure S1. Effect of compounds on SARS-CoV-2 Spike Vpp infection in Huh7 cells. Cells were pre-treated with different concentrations of the compounds for 1 h and transduced with Vpp for 1 h in the presence of the compounds in the media. Infectivity was measured using luciferase assay 3 days post infection. The values represent the means ± standard deviation (SD) of data from three independent experiments. *, $P<0.05$; **, $P<0.01$; and ***, $P<0.001$ compared with controls (n=3).

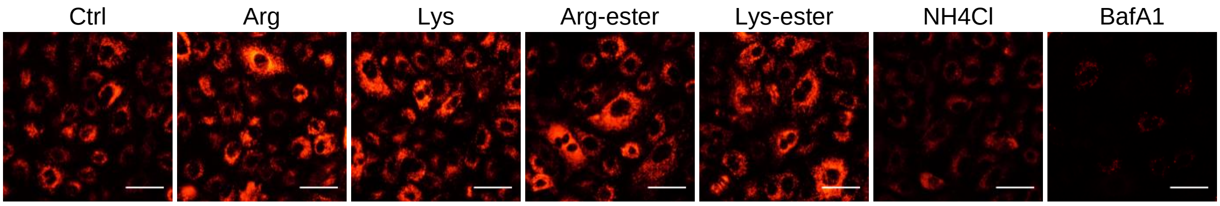


Supplementary Figure S2. LysoTracker Red staining of A549 cells following compound treatment for 1 h. Cells were treated with the compounds (10 mM), $NH_4Cl$ (20 mM) or bafilomycin A1 (40 nM) for 1 h and stained with 75 nM LysoTracker Red for 15 min. Acidic vacuoles were observed using fluorescence microscopy. Scale bar = 50 μM.
